# P02.51. Acupuncture treatment for hospitalized patients on anticoagulant therapy- a safety study

**DOI:** 10.1186/1472-6882-12-S1-P107

**Published:** 2012-06-12

**Authors:** C Miller, B Hopperstad, P Johnson, J Dusek

**Affiliations:** 1 Abbott Northwestern Hospital, Penny George Institute for Health & Healing, Minneapolis, USA; 2 Center for Healthcare Innovation, Allina Hospitals and Clinics, Minneapolis, USA

## Purpose

As acupuncture becomes increasingly available in allopathic medical settings, healthcare providers raise concerns about patient safety. One such concern is excessive bleeding. Although “bleeding” and “bloodletting” are at times recognized by practitioners of Chinese Medicine as therapeutic techniques, bleeding has been described in the modern acupuncture research literature as an adverse event. The purpose of this study is to examine the incidence of bleeding at acupuncture points in hospitalized patients on anticoagulants.

## Methods

Data were obtained from electronic medical records for 1273 inpatient acupuncture treatments occurring at one large Midwestern hospital between January and June 2010. We examined data for 350 treatments received by 229 patients on warfarin who also had an INR drawn the same day. INR values were dichotomized at the 90th percentile as high (>2.3) or low (< 2.3). Means and percents were compared by INR level. Analysis included chi-square, t-tests and Wilcoxon rank-sum tests.

## Results

The sample included 106 males and 123 females ages 29 to 95 (mean = 64.4 years; SD=11.6). Overall, 350 acupuncture treatments were observed (median per patient = 1; Q1,Q3 = 1,2). Few significant differences in patient characteristics were detected by INR classification. Of 350 treatments, 14.6% (n=51) had bleeding noted. Cleanup with Q-tip occurred for 98% (n = 50). Percent with bleeding noted was no different between those with high INR and those with low INR (14.3% vs. 14.6%; p = 0.96).

## Conclusion

Minimal bleeding was noted at acupuncture points in patients on anticoagulant therapy. Moreover, incidence of bleeding did not differ by INR level for this sample. Our findings suggest that acupuncture can be used safely for patients on anticoagulant therapy with INR values in the therapeutic range.

